# Supplementary material for: Dissection of Gαs and Hedgehog signaling crosstalk reveals therapeutic opportunities to target adenosine receptor 2b in Hedgehog-dependent tumors
Source: bioRxiv. 2025 Feb 27:2025.02.21.639530. Preprint. [Version 1] doi: 10.1101/2025.02.21.639530 (PMC11888225; doi:10.1101/2025.02.21.639530)
Supplement: Supplement 1 [file NIHPP2025.02.21.639530v1-supplement-1.pdf]

bioRxiv preprint doi: https://doi.org/10.1101/2025.02.21.639530; this version posted February 27, 2025. The copyright holder for this preprint (which was not certified by peer review) is the author/funder. This article is a US Government work. It is not subject to copyright under 17 USC 105 and is also made available for use under a CC0 license.

Figure S1. **A-** PCA plot showing clustering of the different mice by their gene expression profile in bulk mRNA-seq. **B-** Heat map from bulk mRNA-sequencing data comparing the fold change (log2FC) increased in BCC-related genes in the indicated mice. **C-** Functional analysis of pathways differentially regulated in BCC mouse models with respect to control mice. Generated using profile bulk mRNA-seq data with Ingenuity Pathway Analysis software (IPA, Ingenuity Systems). **D-** Expression of cluster-defining genes projected into UMAP of cell clusters for all mice (Fig. 2C). **E-** Relative expression of the indicated genes in each cell compartment for each mouse genotype from single-cell RNAseq data. **F-** Left: expression of *Gnas* projected into a UMAP of each mouse model. Right: violin plot depicting the expression of *Gnas* for individual cells in each compartment in control (Ctrl) and *Gnas*-eKO mice.

bioRxiv preprint doi: https://doi.org/10.1101/2025.02.21.639530; this version posted February 27, 2025. The copyright holder for this preprint (which was not certified by peer review) is the author/funder. This article is a US Government work. It is not subject to copyright under 17 USC 105 and is also made available for use under a CC0 license.

Figure S2. Amplified version of Figure 5A. Circos plot representing BCC GPCR expression in mice, showing class, gene name, Gα coupling, fold change (FC) and expression level (Log2TPM) in bulk RNAseq, level of expression in single-cell RNA sequencing (Log2Counts) in interscale (iSC) and BCCA clusters.

Figure S3. **A-** Expression of the indicated GPCRs projected into a UMAP of each mouse model. UMAP of cell clusters for all mice (Fig. 2C) and names of cell clusters is included for reference. **B-** Schematic representation of the mouse model used to target SMO deletion and PKI expression to the skin. Images on the right show representative H&E staining of tail skin from the indicated mice 34 days after induction. + indicates transgene is present, -/+ indicates heterozygous knockout and -/- indicates homozygous knockout. **C-** Quantification of tumor burden per mouse in H&E tail skin stainings in PKI^{+} mice with *Smo* heterozygous (+/-) or homozygous (-/-) knockout. Graphs show mean ± SEM. N= 10 SmoF +/- mice, N= 14 SmoF -/- mice, t-test. **D-** qRT-PCR analysis of mRNA expression of indicated markers in tail epidermal keratinocytes isolated from PKI^{+} mice with *Smo* heterozygous (+/-) or homozygous (-/-) knockout 34 days after induction. Graphs show mean ± SEM. N= 4 SmoF +/- mice, N= 4 SmoF -/- mice, t-test. SmoF +/- are used as control (Ctrl).

Figure S4. **A-** Schematic representation of the mouse model used to target *Adgra2* deletion to the skin. **B-** Validation of effective *Adgra2* deletion by PCR in the indicated mice. **C-** Representative H&E staining of tail skin from the indicated mice 26 weeks after induction. **D-** Western blot analysis of expression of Gli3-Repressor and expression of doxycycline (Dox)-inducible GPR161-WT and inactivating mutation GPR161-V129E constructs in NTERT human keratinocytes. Molecular weight markers (kDa) are indicated on the left. **E-** Transcriptional activity of CREB measured by CRE-luciferase assay in HEK293 cells transfected with the indicated constructs. Graphs show mean ± SEM.

bioRxiv preprint doi: https://doi.org/10.1101/2025.02.21.639530; this version posted February 27, 2025. The copyright holder for this preprint (which was not certified by peer review) is the author/funder. This article is a US Government work. It is not subject to copyright under 17 USC 105 and is also made available for use under a CC0 license.

Fig S5. **A-** Western blot analysis of SMO and Keratin 17 expression in a SmoM2 cell line generated from BCC tail tumors from SmoM2 mice. Molecular weight markers (kDa) are indicated on the left. **B-** Western blot analysis of E-Cadherin and Keratin 17 expression in SmoM2 cell culture model after 30, 60, and 90 days (D) in culture. Molecular weight markers (kDa) are indicated on the left. **C-** Population doubling graph of SmoM2 cells over 60 days with best line fit regression curve and average doubling time. **D-** IF of SmoM2 cells with magnified field showing the cilia marker ARL13B (red) and ciliary localization of SMO (green). SmoM2 mice and cells express SMO fused to YFP protein. **E and F-**

bioRxiv preprint doi: https://doi.org/10.1101/2025.02.21.639530; this version posted February 27, 2025. The copyright holder for this preprint (which was not certified by peer review) is the author/funder. This article is a US Government work. It is not subject to copyright under 17 USC 105 and is also made available for use under a CC0 license.

Representative images from H&E staining and quantification of tumor volume of SmoM2 xenograft tumors from subcutaneous injection of SmoM2 keratinocytes in the flank of NOD/SCID mouse, 35 days after injection. **G-** Heat map from single cell RNAseq data from the indicated cell groups comparing the relative expression of the Gαs-coupled GPCRs among mouse models.

Fig S1

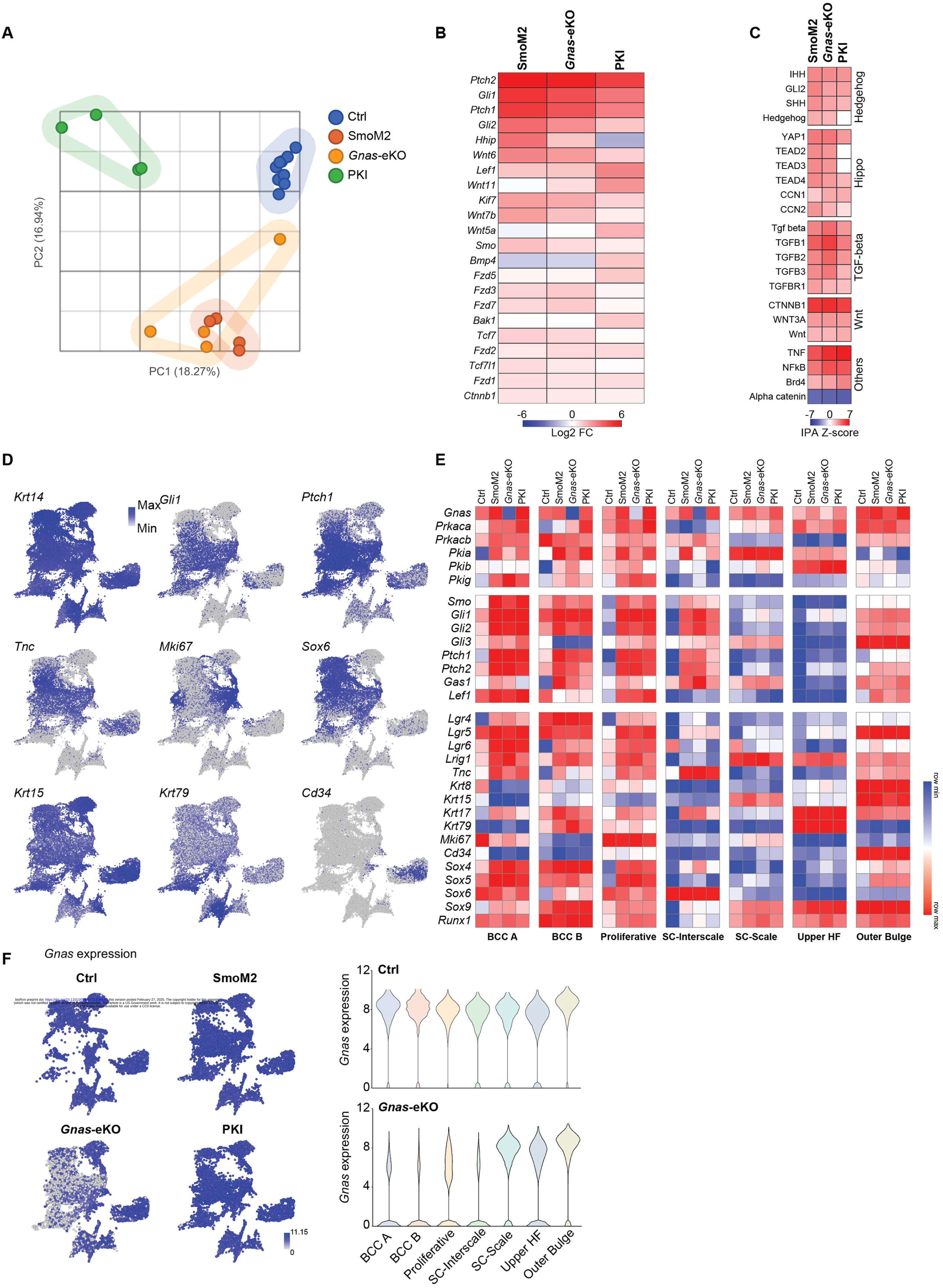

bioRxiv preprint doi: https://doi.org/10.1101/2025.02.21.639530; this version posted February 27, 2025. The copyright holder for this preprint (which was not certified by peer review) is the author/funder. This article is a US Government work. It is not subject to copyright under 17 USC 105 and is also made available for use under a CC0 license.

Fig S2

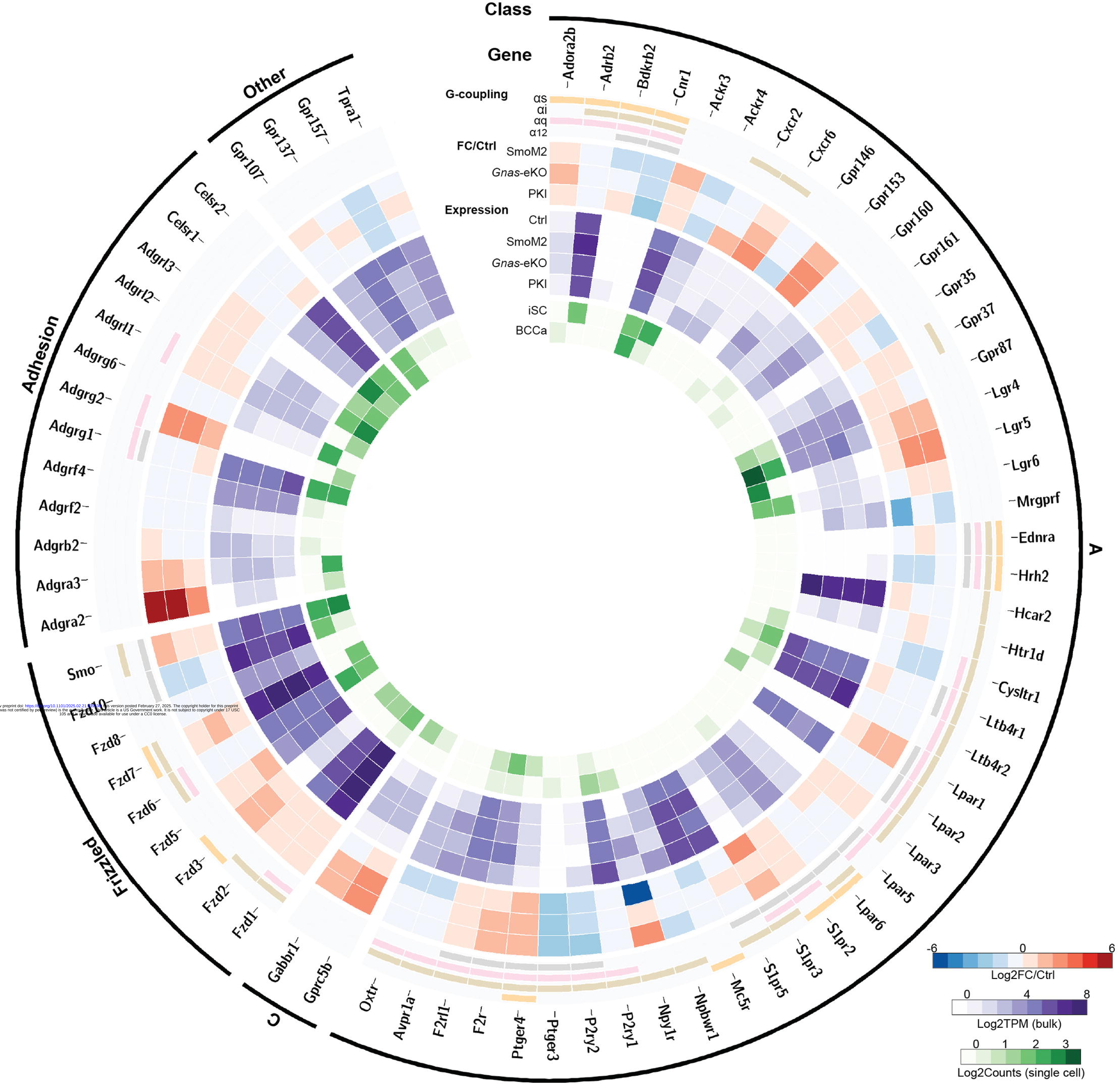

bioRxiv preprint doi: https://doi.org/10.1101/2025.02.21.639530; this version posted February 27, 2025. The copyright holder for this preprint (which was not certified by peer review) is the author/funder. This article is a US Government work. It is not subject to copyright under 17 USC 105 and is also made available for use under a CC0 license.

Fig S3

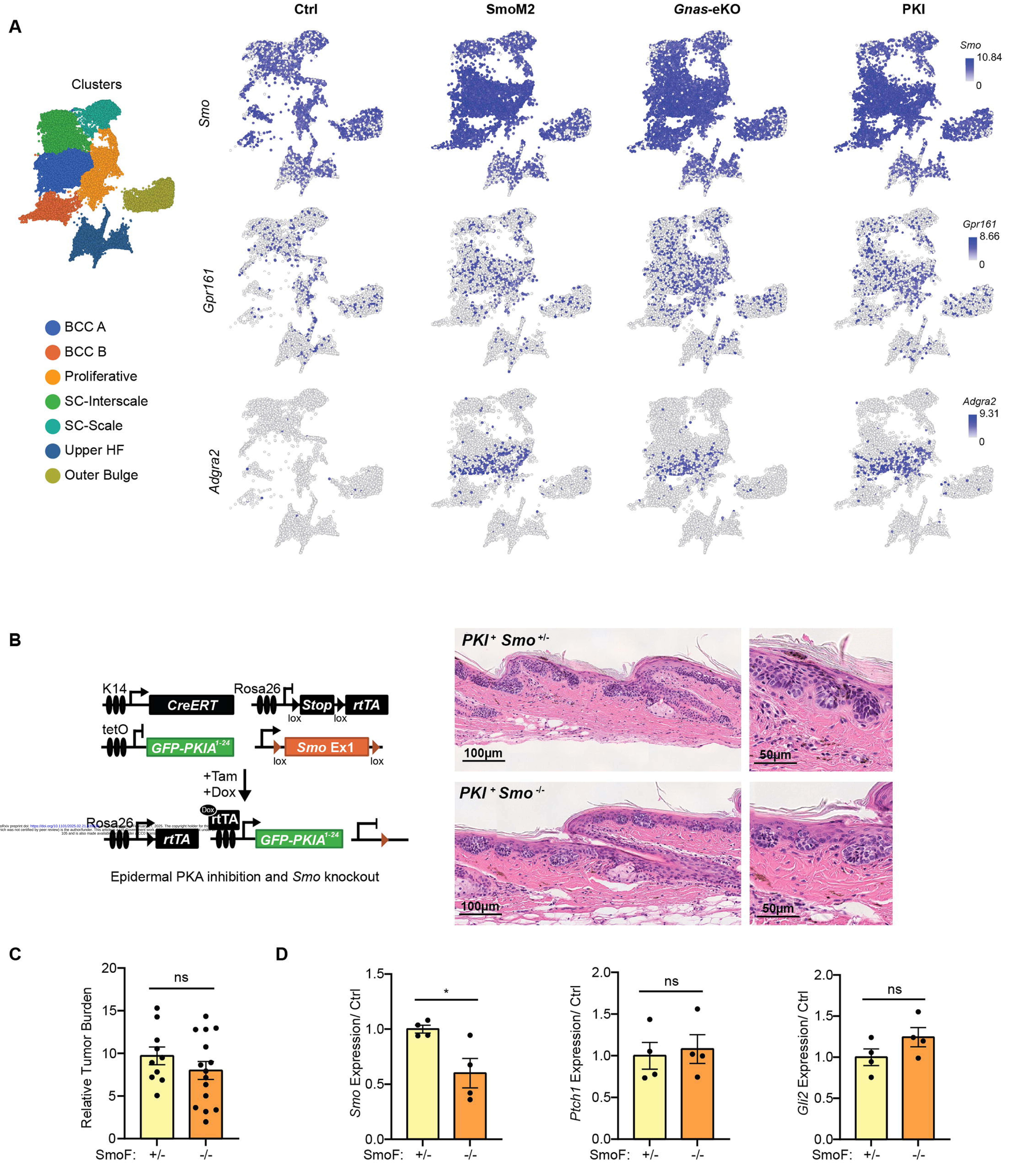

bioRxiv preprint doi: https://doi.org/10.1101/2025.02.21.639530; this version posted February 27, 2025. The copyright holder for this
(which was not certified by peer review) is the author/funder. This article is a US Government work. [illegible]
105 and is also made available [illegible] under a CC0 license.

Fig S4

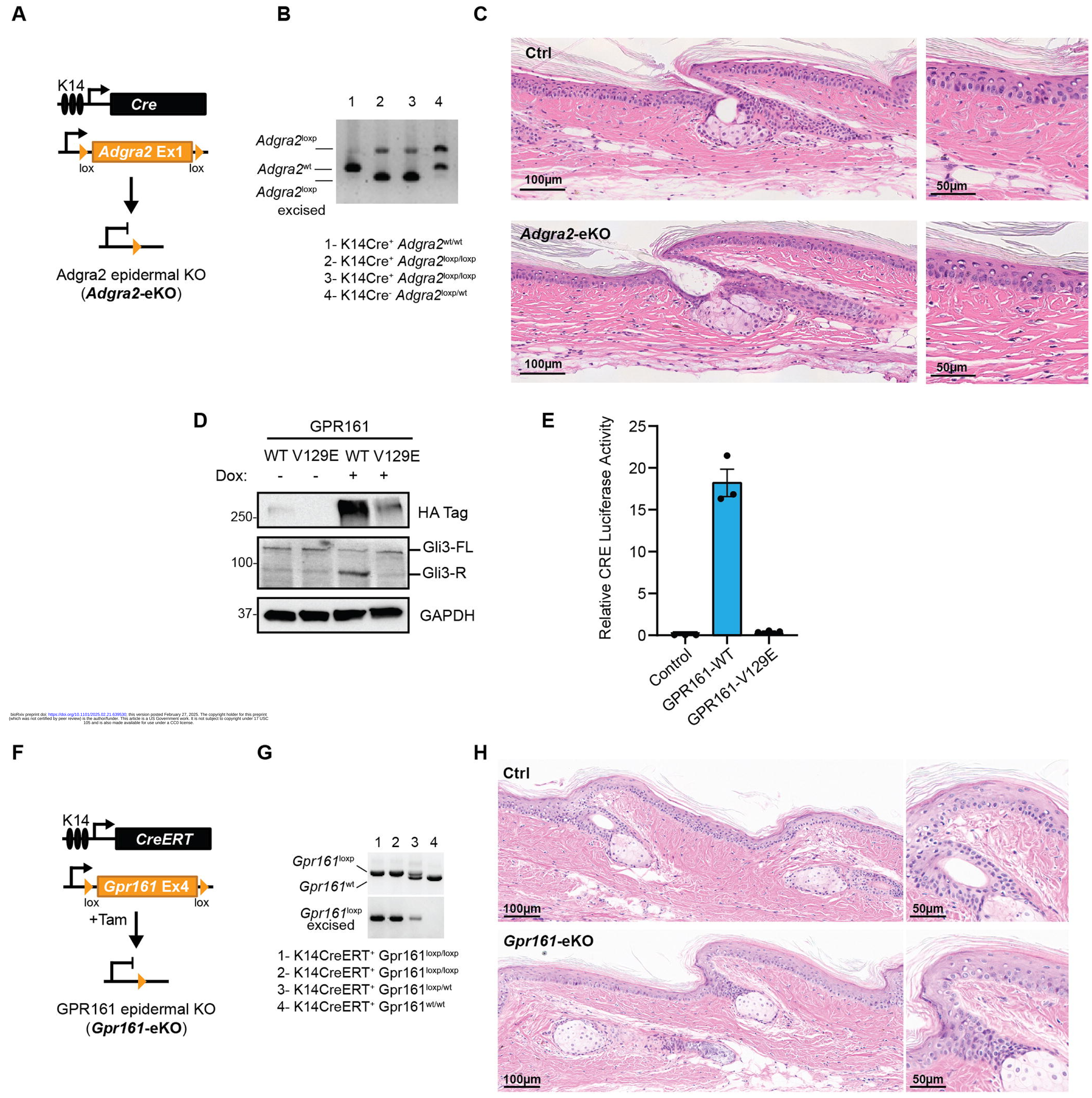

bioRxiv preprint doi: https://doi.org/10.1101/2025.02.21.639530; this version posted February 27, 2025. The copyright holder for this preprint (which was not certified by peer review) is the author/funder. This article is a US Government work. It is not subject to copyright under 17 USC 105 and is also made available for use under a CC0 license.

Fig S5

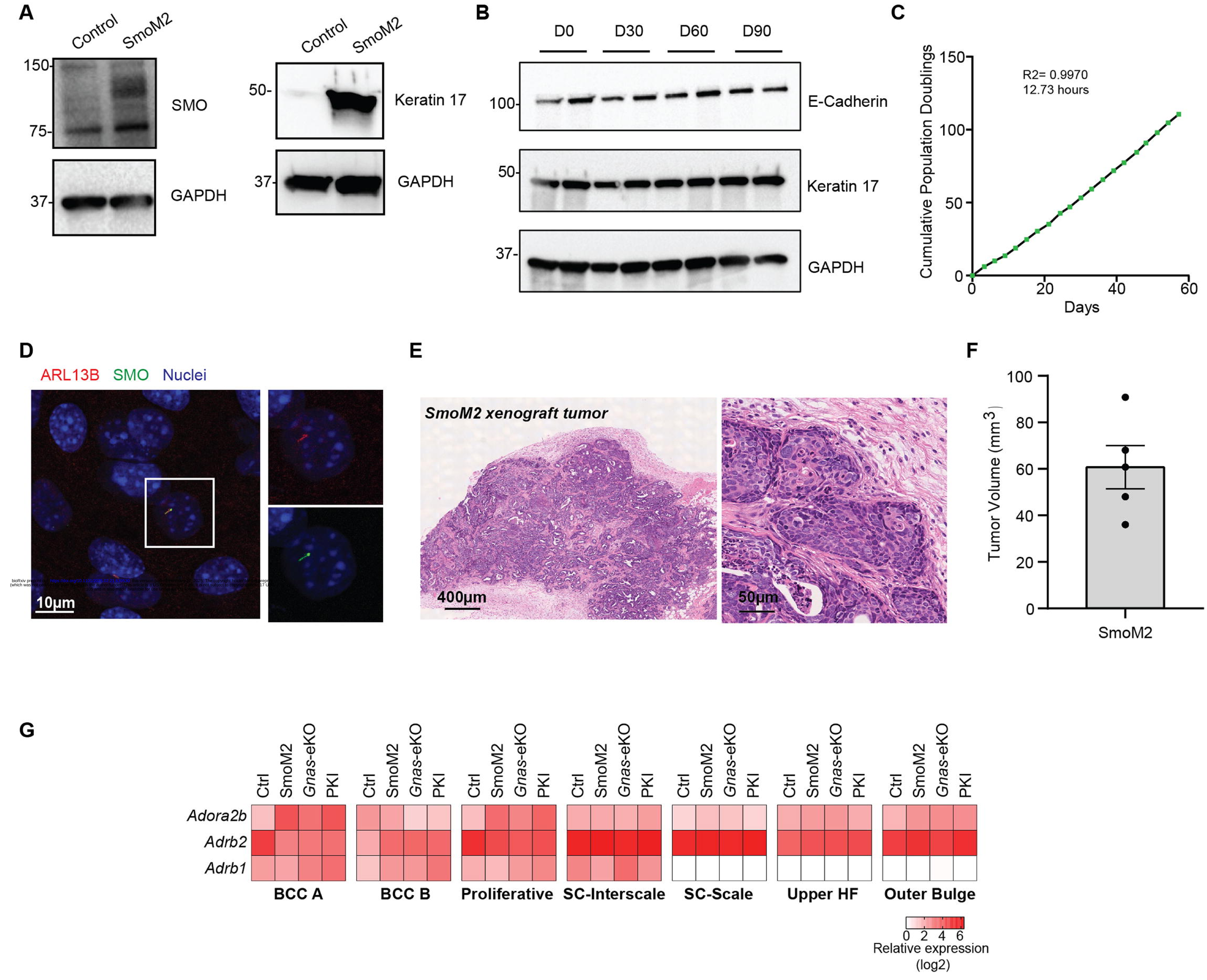
A
Control
SmoM2
150-
75-
SMO
37-
GAPDH
Control
SmoM2
50-
Keratin 17
37-
GAPDH
B
D0
D30
D60
D90
100-
E-Cadherin
50-
Keratin 17
37-
GAPDH
C
Cumulative Population Doublings
R2= 0.9970
12.73 hours
150
100
50
0
0
20
40
60
Days
D
ARL13B SMO Nuclei
10µm
E
SmoM2 xenograft tumor
400µm
50µm
F
Tumor Volume (mm3)
100
80
60
40
20
0
SmoM2
G
Ctrl
SmoM2
Gnas-eKO
PKI
Adora2b
Adrb2
Adrb1
BCC A
BCC B
Proliferative
SC-Interscale
SC-Scale
Upper HF
Outer Bulge
0 2 4 6
Relative expression
(log2)
